# Efficacy of *Wolbachia*-based mosquito control: Predictions of a spatially discrete mathematical model

**DOI:** 10.1371/journal.pone.0297964

**Published:** 2024-03-04

**Authors:** David Dye, John W. Cain

**Affiliations:** Department of Mathematics, Harvard University, Cambridge, MA, United States of America; Texas Christian University, UNITED STATES

## Abstract

*Wolbachia* is an endosymbiont bacterium present in many insect species. When *Wolbachia*-carrying male *Aedes aegypti* mosquitoes mate with non-carrier females, their embryos are not viable due to cytoplasmic incompatibility. This phenomenon has been exploited successfully for the purpose of controlling mosquito populations and the spread of mosquito-borne illnesses: *Wolbachia* carriers are bred and released into the environment. Because *Wolbachia* is not harmful to humans, this method of mosquito control is regarded as a safer alternative to pesticide spraying. In this article, we introduce a mathematical framework for exploring (i) whether a one-time release of *Wolbachia* carriers can elicit a sustained presence of carriers near the release site, and (ii) the extent to which spatial propagation of carriers may allow them to establish fixation in other territories. While some prior studies have formulated mosquito dispersal models using advection-reaction-diffusion PDEs, the predictive power of such models requires careful ecological mapping: advection and diffusion coefficients exhibit significant spatial dependence due to heterogeneity of resources and topography. Here, we adopt a courser-grained view, regarding the environment as a network of discrete, diffusively-coupled “habitats”—distinct zones of high mosquito density such as stagnant ponds. We extend two previously published single-habitat mosquito models to multiple habitats, and calculate rates of migration between pairs of habitats using dispersal kernels. Our primary results are quantitative estimates regarding how the success of carrier fixation in one or more habitats is determined by: the number of carriers released, sizes of habitats, distances between habitats, and the rate of migration between habitats. Besides yielding sensible and potentially useful predictions regarding the success of *Wolbachia*-based control, our framework applies to other approaches (e.g., gene drives) and contexts beyond the realm of insect pest control.

## Introduction

### Mosquito and *Wolbachia* Biology

Mosquito-borne illnesses present a major public health threat. According to World Health Organization estimates, in 2021, there were approximately 247 million cases of malaria worldwide, and the annual number of dengue fever cases now exceeds 100 million. Common methods for controlling mosquito populations and slowing the spread of disease include the use of mosquito nets, repellents, and insecticide sprays.

Within the past two decades, a very different and promising method of mosquito control has generated considerable interest: the use of *Wolbachia* bacteria. *Wolbachia* is an endosymbiont bacterium present in many insect species [1], and is not harmful to humans or the environment [2]. When infected with *w*Mel *Wolbachia* bacteria, *Aedes aegypti* mosquitoes have significantly reduced transmission rates for dengue fever as compared to wild-type *Aedes aegypti* [3–5]. *Wolbachia* additionally reduces the transmission rates for other mosquito-borne viruses such as yellow fever, chikungunya, and Zika virus [6]. Importantly, if a male carrying *w*Mel *Wolbachia* mates with a female non-carrier, their embryos are inviable due to cytoplasmic incompatibility (CI) [3, 7, 8]. This means that *Wolbachia*-carrying males are effectively sterile if they mate with non-carrier females, suggesting a natural control strategy: breed *Wolbachia*-carrying mosquitoes in a laboratory, and release them into a habitat. Such a release may be regarded as “successful” if the carriers establish long-term fixation within the habitat, ideally with carriers being at least as prevalent as non-carriers. The *Wolbachia*-based method of mosquito control was carried out successfully in Cairns, Australia in 2011 [3, 9], leading to reduced dengue transmission [10]. These encouraging results suggest that release of carrier mosquitoes could mitigate disease transmission in other parts of the world.

To predict whether *Wolbachia* infections are successful, researchers have developed various models to represent infection fixation and spread [2, 11–15]. In this article, we propose a new method for modeling infection spread, regarding mosquito habitats (defined later) as a spatially-discrete network, and using dispersal kernels to describe rates of diffusion between pairs of habitats. We explain how to use our framework to predict whether the deliberate introduction of *Wolbachia*-infected mosquitoes in one habitat can achieve fixation not only near the release site, but also in other habitats.

### Importance of spatial structure

Prior studies (see, for example, [14, 16–18]) have proposed advection-reaction-diffusion partial differential equations (PDEs) to model the spatiotemporal dynamics of mosquito dispersal, and the sorts of dynamical behaviors that such models predict (e.g., traveling waves of invasion if certain conditions are met). In the absence of strict simplifying assumptions (e.g., homogeneous density of resources, no spatial dependence of advection and diffusion coefficients, *et cetera*), these models are intractable analytically, which is not to suggest that they do not elicit useful predictions. Still, one cannot ignore the fact that mosquito dispersal is influenced by the heterogeneity of the landscape, and in the resources that support their breeding. The importance of spatial heterogeneity is discussed in [3], which includes some relevant and compelling figures related to a successful release of *Wolbachia* carriers in northeastern Australia, and in the follow-up study of [19]. Given a particular geographic region of interest, ecological mapping studies could potentially yield data informing the spatial dependence of advection and diffusion coefficients appearing in PDE models. Indeed, in [11], the authors model spatial and temporal dependence of diffusion coefficients in a reaction-diffusion model for an *A. aegypti* invasion. It is unclear whether the time, effort and expense of ecologically mapping a single geographic region is worthwhile, in the hopes that a PDE model incorporating spatial heterogeneity will make useful predictions.

In this article, we address the question of whether *Wolbachia* release can succeed, but *without* involving models that might necessitate fine-grained, high-spatial resolution ecological mapping. Instead, we acknowledge spatial heterogeneity by regarding the landscape as a coarse-grained, discrete structure of *habitats*—regions of high mosquito density relative to surrounding areas and with the sorts of resources (e.g., standing water) needed for breeding. Dispersal of mosquitoes between distinct habitats depends upon variety of factors, the most important being the distances between the habitats and the sizes (later interpreted in terms of carrying capacities) of the habitats. By adopting this framework, we eliminate the need to consider models that are targeted to specific geographic locations, and focus attention on the most essential factors that determine whether *Wolbachia* carriers can spread from one region to another.

### Organization of this article

The remainder of this article is organized as follows. In the Mathematical Modeling section, we recall two previously-published differential equation models for mosquito populations. The first is a minimal model which tracks only two subpopulations: carriers and non-carriers of *Wolbachia*, without regard to life stage or sex. The second model tracks mosquitoes according to life stage, carrier status, and sex. Subsequently, we introduce multi-habitat versions of those models, as a step towards being able to test conditions under which release of carriers in one habitat might elicit spread to other habitats. In the Estimating Model Parameters section, we discuss estimation of model parameters for the above models, with particular emphasis on the rates of diffusion between distinct habitats. Using dispersal kernels, we are able to calculate the effective diffusion coefficient associated with a given pair of habitats, as well as the dependence of the diffusion coefficient upon the distance separating the habitats. In the Numerical Simulations section, we survey results of our numerical simulations of multi-habitat models, with diffusion coefficients estimated from two particular choices of dispersal kernel. Importantly, our key results are qualitatively identical regardless of which population model is adopted (i.e., Model 1 or Model 2 from the Mathematical Modeling section) or which dispersal kernel is adopted (i.e., the log-normal or exponential kernels from the Estimating Model Parameters section). There are three typical outcomes following a single-habitat release of carriers: (i) failure to establish carrier presence in any habitat; (ii) sustained presence of carriers only in the region where the release occurred; or (iii) sustained presence of carriers in multiple habitats, including the one where the release occurred. In the Discussion section, we comment on the circumstances that can lead to each of these outcomes, focusing on conditions that favor the spread of *Wolbachia* to multiple habitats.

## Mathematical modeling

One of our central goals is to explain how ordinary differential equation (ODE) models can be used to predict whether release of Wolbachia carrier mosquitoes in one habitat can lead to fixation of carriers in multiple habitats. To this end, we proceed in stages. We begin by recalling two single-habitat models for the spread of Wolbachia: a minimal model that subdivides mosquitoes only according to carrier status, and a more detailed model that distinguishes mosquitoes by sex, life stage, and carrier status. Next, we adapt the single-habitat models to track mosquito populations in *N* ≥ 2 diffusively coupled habitats. The resulting continuous-time, discrete-space ODE models are the focal point of our analysis and simulations; however, we will briefly mention the reaction-diffusion PDEs models one obtains if the spatial domain were continuous. We also discuss parameter estimates for these models, with emphasis on the use of insect dispersal kernels to estimate the strength of diffusive coupling between distinct habitats.

### Single-habitat models

We recall two models for the spread of Wolbachia in mosquito populations. As we shall demonstrate, our primary findings regarding Wolbachia dispersal do not depend upon which of these models is adopted.

#### Model 1: Hu et al. (2021)

The first model, due to Hu et al. [2], is presented as a system of two ODEs that track the proportion of mosquitoes that carry Wolbachia, without accounting for sexes and life stages of mosquitoes. The simplicity of the Hu et al. model makes it analytically tractable for our initial explorations involving dispersal between distinct habitats. The equations are given by

$$\begin{array}{c} \begin{array}{cc} \frac{dx}{dt} & =\left(b_{I}-\delta_{I}\right)x-dx\left(x+y\right)\\ \frac{dy}{dt} & =b_{U}y\frac{y}{x+y}-\delta_{U}y-dy\left(x+y\right), \end{array} \end{array}$$ (1)

where *x* represents the population of Wolbachia-infected mosquitoes, and *y* represents the population of uninfected mosquitoes. The parameters *b*_*I*_, *b*_*U*_, *δ*_*I*_ and *δ*_*U*_ are rate constants associated with (b)irths and (d)eaths of (I)nfected and (U)ninfected mosquitoes. The parameter *d* is a density-dependent death rate and may be re-expressed in terms of the environment’s carrying capacity and the other parameters. As explained in [2], in order to avoid biologically meaningless predictions, the parameters are restricted to obey

$$\begin{array}{c} b_{I}>d+\delta_{I},\;\;\;\;\;b_{U}>d+\delta_{U},\;\;\;\;\;b_{U}\geq b_{I},\;\;\;\;\;\delta_{I}\geq \delta_{U}. \end{array}$$ (2)

**Fig 1:**
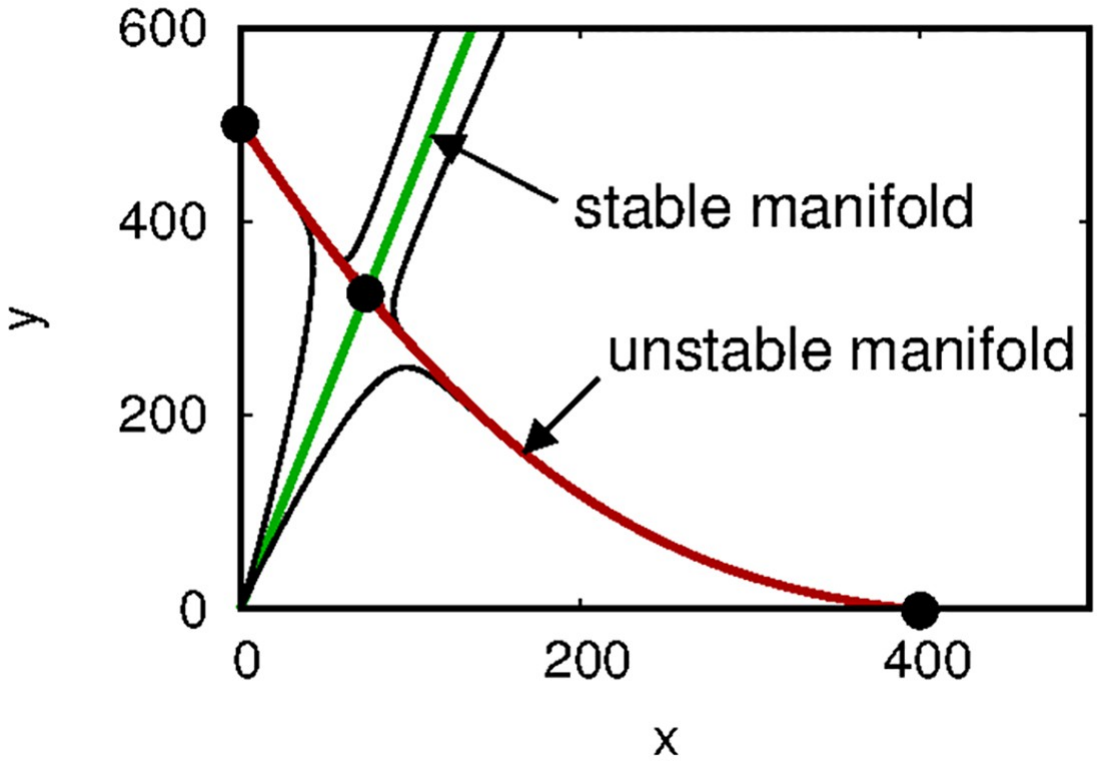
Phase portrait of Eq (1) generated using parameter values *b*_*I*_ = 0.45,*b*_*U*_ = 0.55, *δ*_*I*_ = 0.05, *δ*_*U*_ = 0.048, and *d* = 0.001. Bold dots indicate equilibria. The stable and unstable manifolds of the coexistence equilibrium are shown, along with four other representative trajectories.

If these inequalities are satisfied, then Eq (1) has three equilibria:

$$\begin{array}{c} \left(x_{1},y_{1}\right)=(\frac{b_{I}-\delta_{I}}{d},0)\;\text{and}\;\left(x_{2},y_{2}\right)=(0,\frac{b_{U}-\delta_{U}}{d}) \end{array}$$ (3)

are locally asymptotically stable, and the coexistence equilibrium

$$\begin{array}{c} \left(x_{3},y_{3}\right)=(\frac{\left(b_{U}-\delta_{U}-b_{I}+\delta_{I}\right)\left(b_{I}-\delta_{I}\right)}{b_{U}d},\frac{\left(\delta_{U}+b_{I}-\delta_{I}\right)\left(b_{I}-\delta_{I}\right)}{b_{U}d}) \end{array}$$ (4)

is a saddle. Due to the simplicity of Eq (1), it is straightforward to check that the stable manifold of the coexistence equilibrium lies along the line

$$\begin{array}{c} y=\frac{b_{I}-\delta_{I}+\delta_{U}}{b_{U}-b_{I}+\delta_{I}-\delta_{U}}x \end{array}$$ (5)

in the phase plane. Eq (5) is useful in that it identifies the basins of attraction for the non-coexistence equilibria, thereby providing conditions under which Wolbachia-infected mosquitoes overtake the environment. Fig 1 shows a phase portrait of (1) for a particular choice of parameters. In the next sections, we show various ways of extending this model to represent mosquito dispersal.

#### Model 2: Qu et al. (2018)

The second model, due to Qu et al. [13], is presented as a system of nine ODEs that distinguish *Aedes aegypti* mosquitoes according to sex, life stage, and Wolbachia carrier status among *Aedes aegypti* population density. The equations are

$$\begin{array}{c} \begin{array}{cc} & \frac{dA_{u}}{dt}=(\phi_{u}F_{pu}+v_{u}\phi_{w}F_{pw})(1-\frac{A_{u}+A_{w}}{K})-(\mu_{a}+\psi )A_{u}\\ & \frac{dA_{w}}{dt}=v_{w}\phi_{w}F_{pw}(1-\frac{A_{u}+A_{w}}{K})-(\mu_{a}+\psi )A_{w}\\ & \frac{dF_{u}}{dt}=b_{f}\psi A_{u}-(\sigma +\mu_{fu})F_{u}\\ & \frac{dF_{w}}{dt}=b_{f}\psi A_{w}-(\sigma +\mu_{fw})F_{w}\\ & \frac{dF_{pu}}{dt}=\sigma F_{u}(\frac{M_{u}}{M_{u}+M_{w}})-\mu_{fu}F_{pu}\\ & \frac{dF_{pw}}{dt}=\sigma F_{w}-\mu_{fw}F_{pw}\\ & \frac{dM_{u}}{dt}=b_{m}\psi A_{u}-\mu_{mu}M_{u}\\ & \frac{dM_{w}}{dt}=b_{m}\psi A_{w}-\mu_{mw}M_{w}\\ & \frac{dF_{ps}}{dt}=\sigma F_{u}(\frac{M_{w}}{M_{u}+M_{w}})-\mu_{fu}F_{ps}. \end{array} \end{array}$$ (6)

The nine state variables represent (F)emale, (M)ale and (A)quatic-stage mosquito subpopulations, and subscripts on the state variables indicate the following: *u* = uninfected with Wolbachia; *w* = infected with Wolbachia; *p* = pregnant; and *s* = sterile. The parameter *K* represents the carrying capacity of the aquatic stage mosquitoes in the habitat. Please refer to [13] for a full development of the model equations, and an explanation of the model parameters. In our numerical simulations, we used the parameter values from Table 2.1 of their article.

### Spatially discrete multi-habitat models

Eqs (1) and (6) do not account for mosquito movement and spatial variation in population densities. One could adapt these models into spatially-extended systems, presented as advection-reaction-diffusion equations; this approach is considered in the section A Word on PDE Models below. However, mosquito population densities exhibit considerable spatial variation in accordance with the heterogeneity of topography and resources. From a modeling perspective, this presents a challenge—resource density mapping and estimation of spatially-dependent diffusion coefficients would be a significant undertaking. Instead, we propose a simpler approach in which insects are predominantly concentrated in discrete spatial habitats. By a *habitat*, we have in mind a region in which mosquito population density is high relative to the surrounding areas, e.g., a small stagnant retention pond in an urban setting. If there were two such ponds separated by a wide asphalt highway, the two ponds would be regarded as separate habitats.

For our purposes, it will usually suffice to consider the case of *N* = 2 distinct habitats, say Habitat A and Habitat B. The simplest way to diffusively couple two habitats is to assume that the rate of transfer between habitats is proportional to the difference between the population densities of the two habitats. In the Migration Parameter section, we explain how to compute the rate of diffusion using dispersal kernels under simplifying geographic assumptions. Now, let us introduce multi-habitat versions of Models 1 and 2.

#### Model 1 with multiple habitats

With the preceding paragraph in mind, here is a two-habitat version of Eq (1):

$$\begin{array}{c} \begin{array}{cc} \frac{dx_{A}}{dt} & =\left(b_{I}-\delta_{I}\right)x_{A}-d_{A}x_{A}\left(x_{A}+y_{A}\right)+m\left(x_{B}-x_{A}\right)\\ \frac{dy_{A}}{dt} & =b_{U}y_{A}\frac{y_{A}}{x_{A}+y_{A}}-\delta_{U}y_{A}-d_{A}y_{A}\left(x_{A}+y_{A}\right)+m\left(y_{B}-y_{A}\right)\\ \frac{dx_{B}}{dt} & =\left(b_{I}-\delta_{I}\right)x_{B}-d_{B}x_{B}\left(x_{B}+y_{B}\right)+m\left(x_{A}-x_{B}\right)\\ \frac{dy_{B}}{dt} & =b_{U}y_{B}\frac{y_{B}}{x_{B}+y_{B}}-\delta_{U}y_{B}-d_{B}y_{B}\left(x_{B}+y_{B}\right)+m\left(y_{A}-y_{B}\right), \end{array} \end{array}$$ (7)

where subscripts indicate habitat. We have presumed that the birth and death rate constants are identical for Habitats A and B. The diffusion coefficient *m* has units of time^−1^, and depends at least in part upon the distance between the two habitats. In writing Eq (7), we have assumed that the diffusion coefficient is the same for Wolbachia-infected and uninfected mosquitoes, and that there is no directional bias between the two habitats. (Directional bias can be simulated by using two different diffusion coefficients *m*_*AB*_ and *m*_*BA*_ associated with migration from A to B or vice-versa. However, we shall never have occasion to do so.) We explain how to calculate *m* in the Migration Parameter section. The framework of Eq (7) has been used to model diffusive coupling of two compartments in other contexts; see, for example, Section 6.3.2 of [20] for an illustration of a two-cell Turing instability in a similar system of ODEs.

Eq (7) can be generalized to *N* ≥ 2 distinct habitats. Rather than attempting to write down the most general possible system of equations, we continue to assume that

- the parameters *b*_*I*_, *b*_*U*_, *δ*_*I*_, and *δ*_*U*_ are not habitat-dependent, and
- the diffusion coefficient *m*_*jk*_ associated with movement between habitats *j* and *k* depends upon the distance between those habitats, and there is no directional bias (e.g., due to advection), meaning that *m*_*jk*_ = *m*_*kj*_.

**Henceforth, we shall always adopt these assumptions, even though they could be relaxed very easily.** Indeed, the resulting model equations are general enough to explore the full range of dynamical behaviors that we care about. That being said, the *N*-habitat version of Model 1 is given by

$$\begin{array}{c} \begin{array}{cc} \frac{dx_{j}}{dt} & =\left(b_{I}-\delta_{I}\right)x_{j}-d_{j}x_{j}\left(x_{j}+y_{j}\right)+\sum_{k=1}^{N}m_{jk}\left(x_{k}-x_{j}\right)\\ \frac{dy_{j}}{dt} & =b_{U}y_{j}\frac{y_{j}}{x_{j}+y_{j}}-\delta_{U}y_{j}-d_{j}y_{j}\left(x_{j}+y_{j}\right)+\sum_{k=1}^{N}m_{jk}\left(y_{k}-y_{j}\right), \end{array} \end{array}$$ (8)

where *j* = 1, 2, …, *N* indexes the habitats.

**Fig 2:**
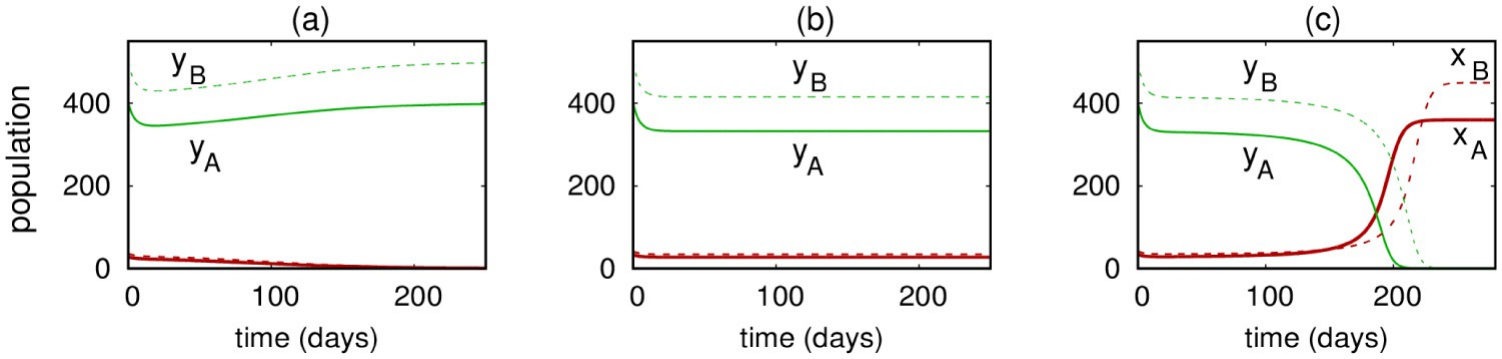
Sample solutions of the two-habitat system Eq (7) using parameters *b*_*I*_ = 0.285,*b*_*U*_ = 0.3, *δ*_*I*_ = 0.079, *δ*_*U*_ = 0.071, *K*_*A*_ = 400,*K*_*B*_ = 500, and *m* = 0.001. Initial populations (*x*_*A*_, *y*_*A*_, *x*_*B*_, *y*_*B*_) are: Left panel (28, 400, 36, 500), middle panel (33.213, 400, 41.516, 500), and right panel (34, 400, 42, 500). Initial conditions for the middle panel lie in the stable manifold of a 4-way coexistence equilibrium.

We do not deem it worthwhile to attempt deep analytical exploration of the Eq (8), e.g., enumerating equilibria, classifying their stability, or describing basins of attraction. However, it merits mentioning that formula (5) for the stable manifold of the coexistence equilibrium in the single-habitat model is helpful for generating intuition concerning basins of attraction for the *N*-habitat model. For instance, for each fixed *j*, one may consider the associated two-variable subsystem of (8) formed by the differential equations for *dx*_*j*_/*dt* and *dy*_*j*_/*dt*. Motivated by Eq (5), it is straightforward to show that if initial conditions satisfy

$$\begin{array}{c} \frac{y_{j}\left(0\right)}{x_{j}\left(0\right)}\;=\;\frac{b_{I}-\delta_{I}+\delta_{U}}{b_{U}-b_{I}+\delta_{I}-\delta_{U}} \end{array}$$ (9)

for each *j* = 1, 2, …, *N*, then the ratios *y*_*j*_(*t*)/*x*_*j*_(*t*) remain constant as *t* increases. Such behavior is illustrated in Fig 2. In this case, the solution trajectory remains confined to the stable manifold of the coexistence equilibrium, approaching that equilibrium as *t* → ∞. The significance of Eq (9) for Model 1 is that it represents a critical ratio of uninfected-to-infected mosquito populations that may determine whether Wolbachia infection persists in each habitat. It also suggests some basic sufficient (but not necessary) conditions for persistence of Wolbachia: if the left-hand side of Eq (9) were less than the right-hand side for each *j*, then the Wolbachia infection persists in each habitat.

#### Model 2 with multiple habitats

Our above process of generalizing a single-habitat model to a multi-habitat model does not depend upon which underlying single-habitat model is used: for each pair of habitats, each *motile* subpopulation of mosquitoes has an associated diffusion term. In generalizing Eq (6) to *N* ≥ 2 habitats, it is not necessary to include diffusion terms in the differential equations for populations in the aquatic life stage (during which migration between habitats is not possible). More precisely, in an *N*-habitat model, the first and third equations in Eq (6) give rise to

$$\begin{array}{c} \begin{array}{cc} & \frac{dA_{u}^{\left(j\right)}}{dt}=(\phi_{u}F_{pu}^{\left(j\right)}+v_{u}\phi_{w}F_{pw}^{\left(j\right)})(1-\frac{A_{u}^{\left(j\right)}+A_{w}^{\left(j\right)}}{K})-(\mu_{a}+\psi )A_{u}^{\left(j\right)}\\ & \frac{dF_{u}^{\left(j\right)}}{dt}=b_{f}\psi A_{u}^{\left(j\right)}-(\sigma +\mu_{fu})F_{u}^{\left(j\right)}+\sum_{k=1}^{N}m_{jk}\left(F_{u}^{\left(k\right)}-F_{u}^{\left(j\right)}\right) \end{array} \end{array}$$ (10)

where superscripts *j* = 1, 2, …, *N* correspond to habitat number. Here, as before, we have simplified matters by assuming that model parameters (such as *ϕ*_*u*_ and *b*_*f*_) are identical across habitats and that *m*_*jk*_ = *m*_*kj*_, though one could easily formulate a more general model, e.g., by replacing *ϕ*_*u*_ with $\phi_{u}^{\left(j\right)}$ and likewise for other parameters. We shall never have occasion to do so and, in all of our multi-habitat simulations of Model 2, we restrict our attention to *N* = 2. Moreover, we will assume that the diffusion coefficients *m*_*jk*_ are identical for each [motile] subpopulation of mosquito.

#### A Word on PDE Models

Other authors have used reaction-diffusion equations to model dispersal of species such as *Aedes aegypti*; see [17] for example. Although our focus is on spatially-discrete models for reasons outlined above, let us take a moment to explore an adaptation of Model 1 to describe population densities in a one-dimensional spatial domain. Let *x* = *x*(*s*, *t*) and *y* = *y*(*s*, *t*) represent population densities (biomass per unit length) of infected and uninfected mosquitoes at position *s* and time *t*. Assuming that the diffusion coefficient *D* does not depend on *s*, Eq (1) with diffusion can be written as the reaction-diffusion system

$$\begin{array}{c} \begin{array}{cc} \frac{\partial x}{\partial t} & =D\frac{\partial^{2}x}{\partial s^{2}}+\left(b_{I}-\delta_{I}\right)x-dx\left(x+y\right)\\ \frac{\partial y}{\partial t} & =D\frac{\partial^{2}y}{\partial s^{2}}+b_{U}y\frac{y}{x+y}-\delta_{U}y-dy\left(x+y\right). \end{array} \end{array}$$ (11)

**Fig 3:**
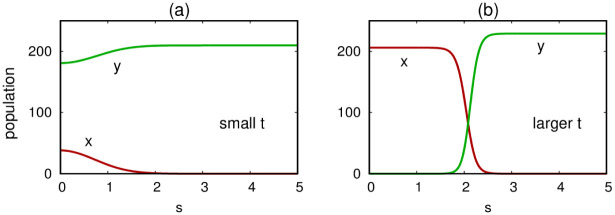
Solutions of Eq (11) for small positive *t* (Panel a) and moderate positive *t* (Panel b) using the parameters from Table 1, *d* = 0.001 and initial conditions described in the main text. A left-to-right traveling wave is established and the infected mosquitoes eventually overtake the entire one-dimensional spatial domain.

Simulations of Eq (11) recreate behaviors that are expected and have been reported previously in other models of insect dispersal [17]. For example, one may generate traveling wave solutions in which an advancing wave of Wolbachia-infected mosquitoes establishes infection fixation in a territory initially saturated by uninfected mosquitoes. Fig 3 illustrates this behavior for a particular set of parameters and using initial density profiles *x*(*s*, 0) = 40exp(−*s*^2^) and *y*(*s*, 0) = 200. The infected mosquitoes invade and overtake the entire one-dimensional landscape. For the parameters appearing in the figure caption, if the amplitude of the initial condition for *x* is reduced below some threshold, a traveling wave is not elicited and only uninfected mosquitoes persist.

## Estimating model parameters

The four types of parameters in the two-habitat model given by Eq (7) are birth rates *b*_*I*_ and *b*_*U*_, density-independent death rates *δ*_*I*_ and *δ*_*U*_, density-dependent death rates *d*_*A*_ and *d*_*B*_, and the migration parameter *m*. As explained in the model definition, the differences in birth rates and death rates between Habitats A and B are assumed to be negligible. However, the density-dependent death rates and the migration parameter are dependent on habitat characteristics.

### Birth and death rates

While the birth and death rate constants for *Aedes aegypti* may depend upon geographic region, Xue et al. established global “baseline” estimates for these parameters, distinguishing between mosquitoes with and without infection by the *w*Mel *Wolbachia* strain [21]. Their parameter estimates, which appear in Table 1, will be used in our numerical simulations.

**Table 1:** Parameter baseline estimates from Xue et al. [21].

Parameter	Symbol	Estimated Value (days^−1^)
Uninfected Birth Rate	*b*_*U*_	0.300
Infected Birth Rate	*b*_*I*_	0.285
Uninfected Density-Independent Death Rate	*δ*_*U*_	0.071
Infected Density-Independent Death Rate	*δ*_*I*_	0.079

### Density-dependent death rate

The density-dependent death rate parameter can be expressed in terms of the carrying capacity of a habitat, together with other model parameters. We shall do so, because we find it more natural to specify carrying capacities for Habitats A and B (call these capacities *K*_*A*_ and *K*_*B*_, respectively) than specifying *d*_*A*_ and *d*_*B*_. The relationship between density-dependent death rates and carrying capacities is obtained by considering equilibrium solutions of Eq (7) in the absence of infected mosquitoes. Setting each derivative to zero, *x*_*A*_ = *x*_*B*_ = 0, *y*_*A*_ = *K*_*A*_, and *y*_*B*_ = *K*_*B*_, one obtains

$$\begin{array}{c} d_{A}=\frac{\left(b_{U}-\delta_{U}\right)K_{A}-m\left(K_{A}-K_{B}\right)}{{\left(K_{A}\right)}^{2}},\;d_{B}=\frac{\left(b_{U}-\delta_{U}\right)K_{B}+m\left(K_{A}-K_{B}\right)}{{\left(K_{B}\right)}^{2}}. \end{array}$$ (12)

Eq (12) can be generalized for the *N*-Habitat Model Eq (8): the density-dependent death rate for the *j*th habitat is given by

$$\begin{array}{c} d_{j}=\frac{1}{K_{j}}(b_{U}-\delta_{U}+\sum_{k=1}^{N}m_{jk}\left(\frac{K_{k}}{K_{j}}-1\right)). \end{array}$$ (13)

#### Bounds on *m* from Model 1 parameters

While the simplicity of Model 1 confers some advantages for numerical and analytical explorations, there is one caveat that we must draw attention to. Without additional restrictions, Eq (12) admits the possibility of negative density-dependent death rates, which is unrealistic on biological grounds. As a step towards establishing inequalities that prevent negative values of *d*_*A*_ and *d*_*B*_, suppose without loss of generality that *K*_*B*_ ≤ *K*_*A*_. Then from Eq (12), we see that *d*_*B*_ > 0 since *b*_*U*_ > *δ*_*U*_. We must guarantee that *d*_*A*_ > 0 as well. For convenience, let

$$\begin{array}{c} a=\frac{K_{B}}{K_{A}} \end{array}$$ (14)

and note that 0 < *a* ≤ 1. Substituting Eq (14) into Eq (12) yields

$$\begin{array}{c} d_{A}=\frac{\left(b_{U}-\delta_{U}\right)K_{A}-m\left(K_{A}-aK_{A}\right)}{{\left(K_{A}\right)}^{2}}=\frac{b_{U}-\delta_{U}-m\left(1-a\right)}{K_{A}}. \end{array}$$ (15)

Requiring the numerator to be positive, we find that *d*_*A*_ > 0 if

$$\begin{array}{c} 0\leq m<\frac{b_{U}-\delta_{U}}{1-a}. \end{array}$$ (16)

Eq (16) is specific to Model 1 and, in all of our subsequent simulations of that model, our parameters are chosen such that the inequality is satisfied.

### Migration parameter

#### Dispersal kernel overview

We use dispersal kernels to calculate the migration parameter *m*. A dispersal kernel is a probability density function (PDF) describing the distribution of a population [22]. One of the best-known uses of dispersal kernels is for estimating seed dispersal patterns [23]. They have also been used by mosquito researchers in mark-release-recapture (MRR) studies to describe mosquito dispersal and estimate the mean distance traveled (*ξ*) of mosquitoes over a study’s experimental period [24–26]. Below, we will express dispersal kernels using both polar and Cartesian coordinates.

Two examples of dispersal kernels used to describe mosquito distributions are the negative exponential PDF and the log-normal PDF [24, 25]. However, other kernels also appear in the literature [27]. A general formula for estimating *m* using a radially-symmetric dispersal kernel is given in Eq (17), and we give examples of calculating *m* using the exponential and log-normal dispersal kernels in the following sections. A derivation of the following equation can be found in the S1 Appendix:

$$\begin{array}{c} m\left(x^{*}\right)=\frac{1}{2\pi t}\int_{0}^{2\pi }\int_{0}^{r^{*}}\frac{p(r\text{cos}\theta +x^{*},r\text{sin}\theta )}{\sqrt{{\left(r\text{cos}\theta +x^{*}\right)}^{2}+{\left(r\text{sin}\theta \right)}^{2}}}\;r\;dr\;d\theta . \end{array}$$ (17)

Here, x* is the distance separating the centers of two circular habitats of radii r*. The given PDF is represented in its Cartesian form, *p*(*x*, *y*). A habitat’s radius is defined as the radius of the disk encompassing a proportion *q* of the total mosquito population, as given by the PDF. The variable *t* is the time elapsed during the experimental period used to estimate the probability density function *p*; see the section Example Estimates of *m* for details.

Eq (17) gives the migration parameter *m* for migration between two circular habitats of radius r* with centers separated by a distance of x*.

#### The negative exponential dispersal kernel

Here, Eq (17) is used to write the formula for *m*(x*) for the negative exponential PDF. The negative exponential PDF expressed in polar coordinates is given by [28]:

$$\begin{array}{c} p_{\text{exp}}\left(r\right)=\frac{1}{\xi }\text{exp}(-\frac{r}{\xi }),\;r\in \left[0,\infty \right). \end{array}$$ (18)

If one defines a habitat’s radius as the radius of the disk enclosing a proportion *q* of mosquitoes in that habitat, then the following equation relates *p*_exp_(*r*), r*, and *q*:

$$\begin{array}{c} q=\int_{0}^{r^{*}}p_{\text{exp}}\left(r\right)\;dr. \end{array}$$ (19)

Substituting Eq (18) into.Eq (19) and solving for r* gives the radius for the negative exponential kernel:

$$\begin{array}{c} r^{*}=-\xi \text{ln}\left(1-q\right). \end{array}$$ (20)

Re-expressing the negative exponential PDF Eq (18) in Cartesian coordinates as

$$\begin{array}{c} p_{\text{exp}}\left(x,y\right)=\frac{1}{\xi }\text{exp}(-\frac{1}{\xi }\sqrt{x^{2}+y^{2}}), \end{array}$$ (21)

we have that Eq (17) yields the following formula for *m* using the negative exponential kernel:

$$\begin{array}{c} m_{\text{exp}}\left(x^{*}\right)=\frac{1}{2\pi t\xi }\int_{0}^{2\pi }\int_{0}^{r^{*}}\frac{r\text{exp}(-\frac{1}{\xi }\sqrt{{\left(r\text{cos}\theta +x^{*}\right)}^{2}+{\left(r\text{sin}\theta \right)}^{2}})}{\sqrt{{\left(r\text{cos}\theta +x^{*}\right)}^{2}+{\left(r\text{sin}\theta \right)}^{2}}}drd\theta . \end{array}$$ (22)

#### The log-normal dispersal kernel

**Fig 4:**
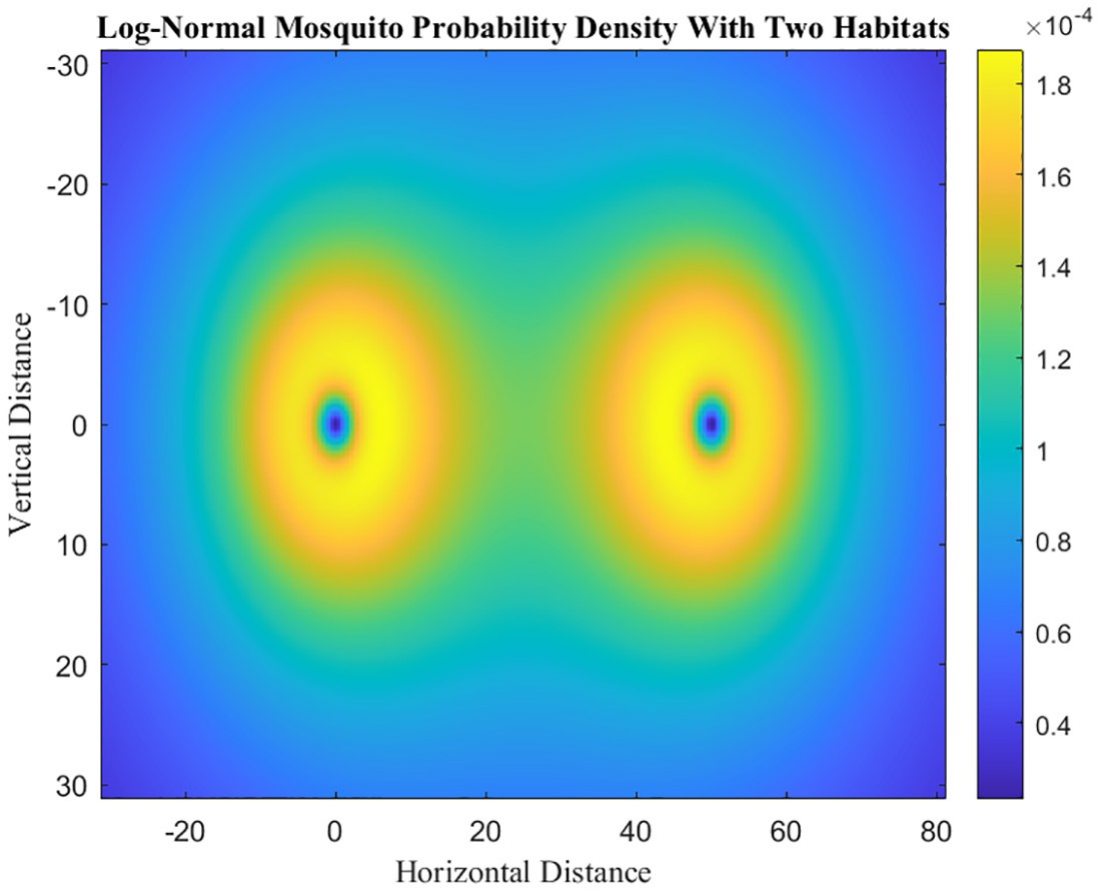
Heat map representing the probability density function of finding a mosquito at a given point in space using log-normal dispersal location kernels in two discrete habitats, assuming the two habitats have the same number of mosquitoes present. The habitats are separated by a distance of 50 meters, and the mean distance traveled (*ξ*) is 75 meters with a standard deviation *σ* of 50 meters.

Now, we will compute *m*(x*) for the log-normal kernel (see also Fig 4). The log-normal dispersal kernel is given by [29]:

$$\begin{array}{c} p_{\text{log}}\left(r\right)=\frac{1}{Sr\sqrt{2\pi }}\text{exp}(-\frac{{\left(\text{ln}\left(r\right)-M\right)}^{2}}{2S^{2}}), \end{array}$$ (23)

where M and S are functions of the mean distance traveled (*ξ*) and standard deviation *σ*:

$$\begin{array}{c} M=\text{ln}(\frac{\xi^{2}}{\sqrt{\xi^{2}+\sigma^{2}}}),\;\;\;\;\;S=\sqrt{\text{ln}(\frac{\xi^{2}+\sigma^{2}}{\xi^{2}})}. \end{array}$$ (24)

Re-expressing the log-normal PDF Eq (23) in Cartesian coordinates,

$$\begin{array}{c} p_{\text{log}}\left(x,y\right)=\frac{1}{\left(\sqrt{2\pi }\right)S\sqrt{x^{2}+y^{2}}}\text{exp}(-\frac{{\left(\text{ln}\left(\sqrt{x^{2}+y^{2}}\right)-M\right)}^{2}}{2S^{2}}). \end{array}$$ (25)

Using Eq (17) as before, we have

$$\begin{array}{c} m_{\text{log}}\left(x^{*}\right)=\frac{1}{{\left(2\pi \right)}^{3/2}St}\int_{0}^{2\pi }\int_{0}^{r^{*}}\frac{r\text{exp}(f(x^{*},r,\theta ))}{{\left(r\text{cos}\theta +x^{*}\right)}^{2}+{\left(r\text{sin}\theta \right)}^{2}}drd\theta , \end{array}$$ (26)

where

$$\begin{array}{c} f\left(x^{*},r,\theta \right)=-\frac{1}{2S^{2}}{\left(\text{ln}\left(\sqrt{{\left(r\text{cos}\theta +x^{*}\right)}^{2}+{\left(r\text{sin}\theta \right)}^{2}}\right)-M\right)}^{2}. \end{array}$$

Eq (26) gives *m*(x*) for migration between habitats of radius r* located a distance of x* apart, assuming the log-normal PDF.

Eq (19) can be used to find the radius r* that encloses a proportion *q* of a habitat’s mosquito population for the log-normal distribution, namely

$$\begin{array}{c} r^{*}=\text{exp}(M-\sqrt{2}S\left(\text{error}\left(1-2q\right)\right)), \end{array}$$ (27)

where error(x) is the inverse error function evaluated at *x*.

#### Example estimates of *m*

Eqs (17), (22) and (26) provide a framework for estimating the migration parameter *m* from different dispersal kernels. In this section, we use those equations to estimate *m* for various distances x*, drawing upon data from [24]. In Yishun and Tampines, Singapore, they fit MRR and genetic data to the exponential kernel and log-normal kernel. Only their MRR data is used for this analysis. In Tampines, Filipović et al. obtained *ξ* = 45.2 meters with a standard deviation of 66.8 meters over 7 days, and in Yishun they obtained *ξ* = 45.2 meters with a standard deviation of 74.4 meters over 7 days [24]. With *t* = 7 days in Eq (17), the *m* values for both locations at various distances are given in Table 2.

**Table 2:** Estimates of *m*(x*) based on *ξ* (mean distance traveled) and standard deviation *σ* from Filipović et al. [24]. Habitat radius encompasses *q* = 95% of mosquitoes, and x* is measured in meters.

Location	Kernel	*ξ*	*σ*	*m*(100)	*m*(150)	*m*(200)	*m*(250)
Tampines	Exponential	45.2	66.8	0.1160	0.0315	0.0062	0.0015
Tampines	Log-normal	45.2	66.8	0.0098	4.55 × 10^−4^	4.27 × 10^−5^	6.20 × 10^−6^
Yishun	Exponential	45.2	74.4	0.1160	0.0315	0.0062	0.0015
Yishun	Log-normal	45.2	74.4	0.0032	9.78 × 10^−5^	6.47 × 10^−6^	6.91 × 10^−7^

The choice of kernel used to estimate *m* is very important. In Table 2, there are orders of magnitude separating *m* values derived from the exponential and log-normal kernels. Therefore, when estimating *m*, it is important to have enough mosquito movement data from the geographic region of interest to determine which kernel best fits the population distribution. In the case of Filipović et al., the exponential kernel provided the best fit of their data [24]. However, other researchers have better results from the log-normal kernel [25]. Additionally, some studies have used time-dependent kernels to model mosquito dispersal [27, 30].

## Numerical simulations

### Basins of attraction

**Fig 5:**
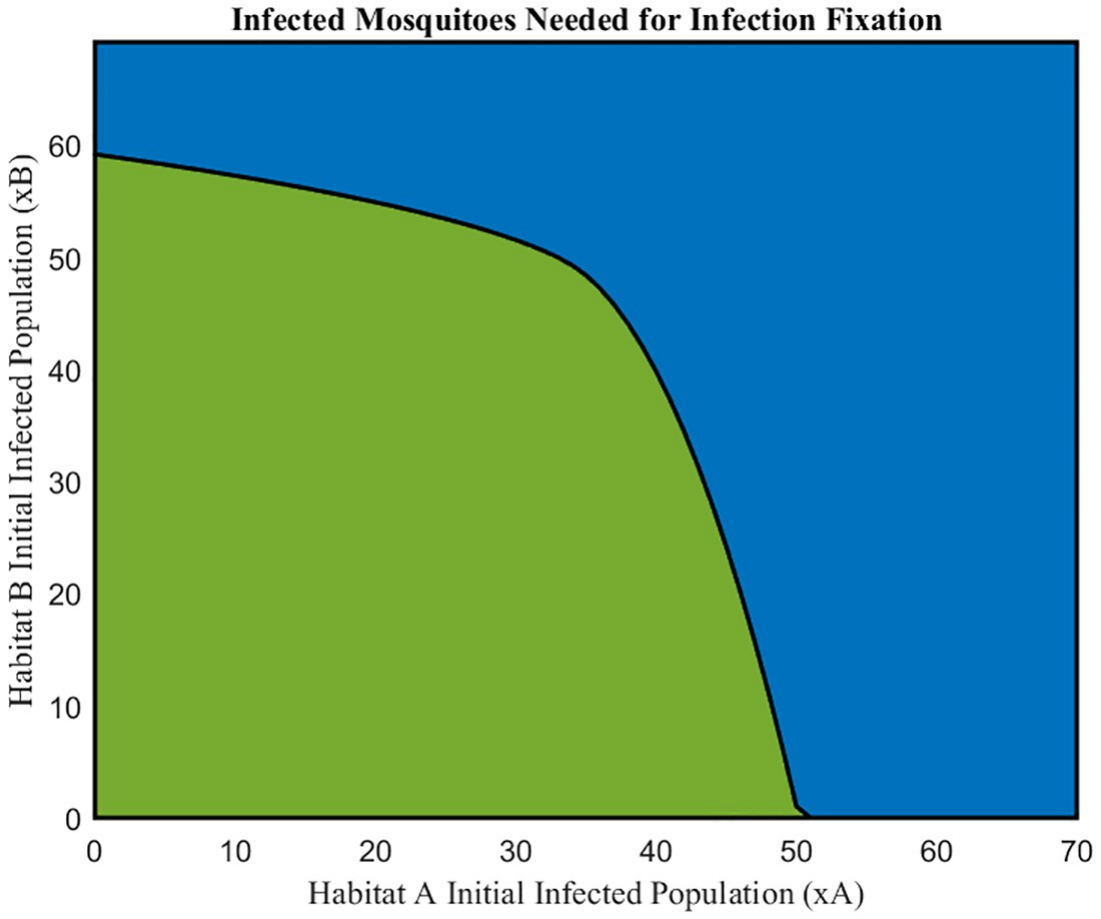
The green (lower left) region represents (*x*_*A*_, *x*_*B*_) starting populations for which the Wolbachia infection eventually disappears, and the blue (upper right) region represents (*x*_*A*_, *x*_*B*_) starting populations that lead to Wolbachia infection fixation. Generated by numerical solution of the two-habitat system Eq (7) using (*y*_*A*_, *y*_*B*_) = (400, 600) and parameters *b*_*I*_ = 0.285, *b*_*U*_ = 0.3, *δ*_*I*_ = 0.079, *δ*_*U*_ = 0.071,*K*_*A*_ = 400, *K*_*B*_ = 600, and *m* = 0.006.

One of our major goals in modeling *Wolbachia*-based control of *Aedes aegypti* is to estimate how many infected mosquitoes must be introduced into a habitat to achieve a sustained infection. In order to address this question using the two-habitat model Eq (7), we fixed all model parameters as well as the initial conditions for uninfected mosquito populations (*y*_*A*_ and *y*_*B*_) and identified the initial conditions for Wolbachia-infected populations (*x*_*A*_ and *x*_*B*_) for which Wolbachia fixation is achieved in both habitats. Fig 5 summarizes the results, illustrating a boundary above which a successful *Wolbachia* invasion is expected to occur. The asymmetry in the figure is caused by the different habitat carrying capacities, *K*_*A*_ and *K*_*B*_.

The intercepts of the curved boundary in Fig 5 are noteworthy, as they indicate the minimum number of infected mosquitoes that must be added to a single habitat to elicit infection fixation in both habitats. Under the conditions used to generate Fig 5, introducing more than 60 infected mosquitoes to Habitat B will cause infection fixation, while introducing more than 51 infected mosquitoes to Habitat A will have the same effect. Since Habitat A has a smaller carrying capacity than Habitat B, this means that introducing *Wolbachia*-infected mosquitoes to the smaller habitat makes it easier to infect the larger habitat for the parameters used in Fig 5.

### Migration-dependent behavior

Steady-state behavior in the two-habitat model Eq (7) is most influenced by two habitat characteristics: migration between habitats and carrying capacities of habitats.

**Fig 6:**
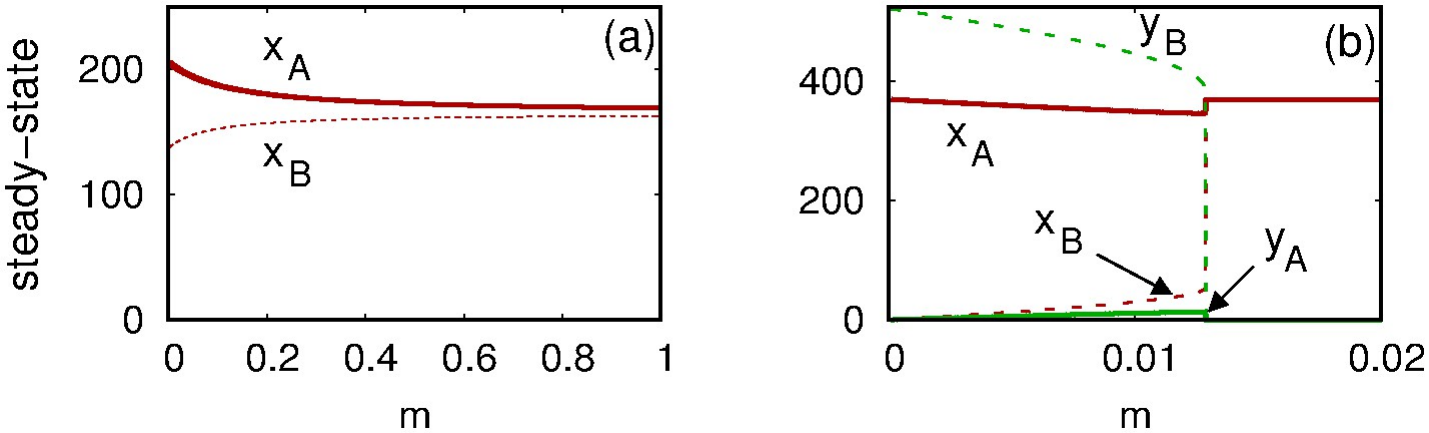
Effect of migration on equilibria. See main text for parameter values and initial conditions, noting that these are different for the two panels. (a) Increasing *m* leads to coalescing of steady-state populations of Wolbachia carriers in Habitats A and B. (b) For small *m* Wolbachia carriers are prevalent in Habitat A only. Increasing *m* leads to fixation of carriers and exclusion of uninfected mosquitoes in both habitats.

Increasing the migration parameter *m* causes linked habitats to behave more similarly. No migration (*m* = 0) means that the habitats have no effect on each other. As *m* increases, the habitats effectively merge. Fig 6(a) shows steady-state values of Wolbachia-infected populations (*x*_*A*_ and *x*_*B*_) for two habitats as *m* is varied but all other parameters held fixed (*b*_*I*_ = 0.285, *b*_*U*_ = 0.3, *δ*_*I*_ = 0.079, *δ*_*U*_ = 0.071, *d*_*A*_ = 0.001, and *d*_*B*_ = 0.0015) and using initial conditions *x*_*A*_(0) = *y*_*B*_(0) = 100 and *x*_*B*_(0) = *y*_*A*_(0) = 0. Note that the two curves in the figure merge as *m* increases.

When the migration parameter *m* is very small, the *Wolbachia* infection may succeed in one habitat despite failing in the other, while increasing *m* can lead to success or failure in both habitats. Such behavior is illustrated in Fig 6(b), using parameters *b*_*I*_ = 0.42, *b*_*U*_ = 0.57, *δ*_*I*_ = 0.05, *δ*_*U*_ = 0.048, and *d*_*A*_ = *d*_*B*_ = 0.001. For *m* = 0, Wolbachia-infected mosquitoes completely overrun Habitat A but are absent in Habitat B. For 0 < *m* < 0.0128, there is a stable equilibrium in which all four mosquito subpopulations are positive. (We caution the reader that the solid and dashed curves are **not** meant to indicate stability or instability—solid curves correspond to Habitat A and dashed curves correspond to Habitat B.) Increasing *m* from zero causes the steady-state infected population to decrease in Habitat A and increase in Habitat B, as indicated by the curves labeled *x*_*A*_ and *x*_*B*_ in the figure. The reverse is true of steady-state uninfected populations: *y*_*B*_ gradually decreases with *m* while *y*_*A*_ (barely visible in the figure) increases. At *m* ≈ 0.128, a bifurcation occurs. For *m* > 0.0128, the uninfected populations *y*_*A*_ and *y*_*B*_ tend to zero, while both *x*_*A*_ and *x*_*B*_ tend to a positive value. (For this parameter set, it happens that *x*_*A*_ and *x*_*B*_ tend to the same value because *d*_*A*_ = *d*_*B*_.)

### Size-dependent behavior

The carrying capacity of habitats also affects the expected steady-state behavior. If a habitat with a large carrying capacity is saturated with non-carriers of *Wolbachia*, a large initial release of carriers is required to offer any hope of a sustained presence of infected mosquitoes.

As shown in the discussion of an upper bound on *m* in the Estimating Model Parameters section, the relative sizes of two interacting habitats can be important. For the two-habitat model, extensive numerical analysis indicates that fewer infected mosquitoes are required to reach infection fixation if the smaller habitat is infected first, since it will reach fixation quickly and then spread to the larger habitat gradually. However, if migration is too small, the infection is unable to spread to the larger habitat.

### Simulations with the *N*-Habitat model for Model 1

For *N* > 2, the *N*-Habitat model can exhibit a variety of behaviors (e.g., fixation of *Wolbachia* carriers in some subset of the habitats), and we do not attempt a comprehensive survey of results that are largely intuitive. We will, however, showcase some interesting cases of the *N*-Habitat model when applied to Eq (1).

**Fig 7:**
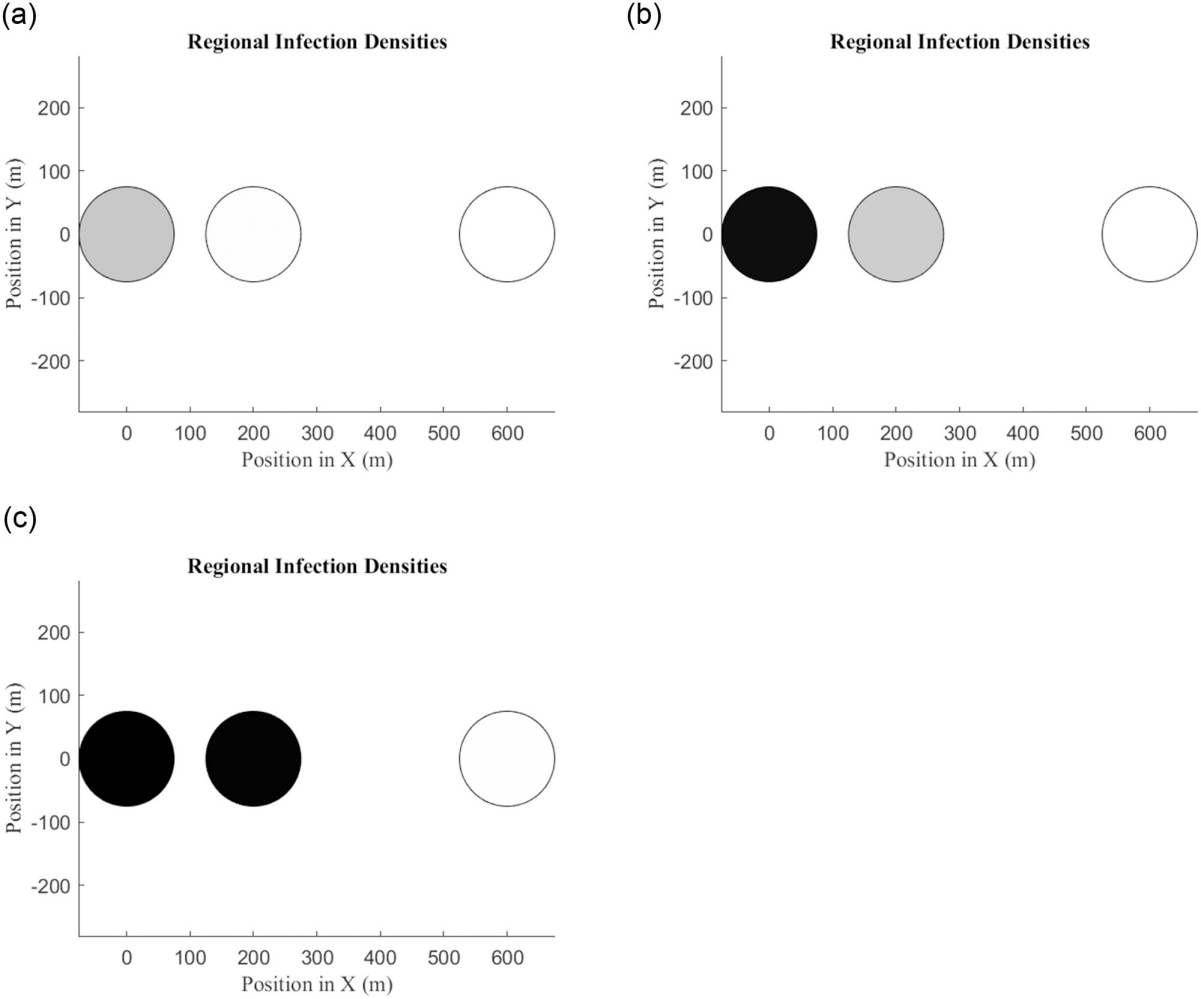
The infection spreads from the leftmost habitat to the middle habitat, but it cannot spread successfully to the rightmost habitat due to the increased distance. Generated with *b*_*I*_ = 0.45, *b*_*U*_ = 0.55, *δ*_*I*_ = 0.05,*δ*_*U*_ = 0.048, *d* = 0.001, and carrying capacities *K*_*a*_, *K*_*b*_ = 300. Going left to right, habitats are separated by 200 and 400 meters, and *m* values for those distances were calculated using the negative exponential kernel with *ξ* = 75, *q* = 0.8 and *t* = 5.

The first example is shown in Fig 7, in which darker colors indicate higher population densities. A series of three collinear habitats, all with the same carrying capacities, are separated by distances increasing from the leftmost habitat. The infection is introduced in the leftmost habitat, is able to infect the middle habitat, but the rightmost habitat is too far away and escapes the Wolbachia infection. This is one instance of a more general behavior exhibited by the model: spread of an infection stalls in regions for which habitats are too sparsely distributed.

**Fig 8:**
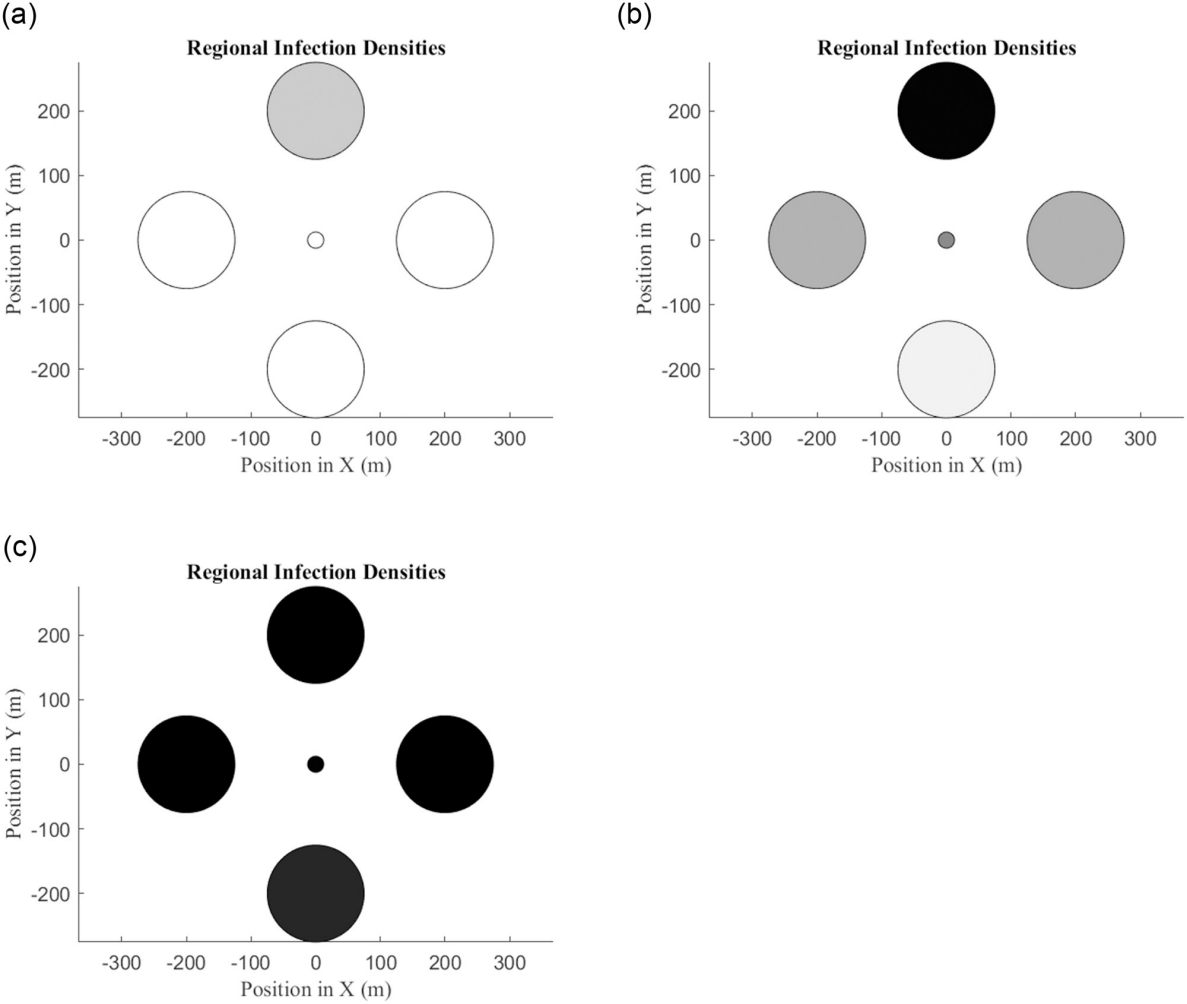
The only way for the infection to reach fixation in this system is if the infected mosquitoes are released into one of the large habitats. Generated with the same parameters as Fig 7. Habitats are separated by 200, 400, or $200\sqrt{2}$ meters.

In the two-habitat system, it is easier to infect the smaller habitat first, after which the infection may spread to the larger habitat. Unfortunately, this is not always the case in the *N*-habitat model. If a small habitat is surrounded by larger habitats, it may be impossible for the infection to ever take hold if it starts from the small habitat. However, a release population of infected mosquitoes that would fail in the small habitat could take over the spatial domain if they are instead released in one of the larger habitats. An example of this is given in Fig 8. The ideal release point of mosquitoes depends on both the sizes and locations of habitats.

### Traveling wave solutions revisited

Prior studies [11, 14] in which PDEs were used to mathematically model mosquito dispersal raised the possibility of the emergence of traveling waves. We illustrated this in Fig 3, simulating a PDE adaptation of Model 1 with a homogeneous spatial domain. The notion of traveling waves does not make sense for our spatially discrete *N*-habitat framework, except in contrived scenarios (e.g., a chain of *N* equally-spaced habitats of identical size and carrying capacities arranged along a straight line). One of our driving forces behind using a spatially-discrete model in the first place was to establish a flexible framework that does not require contrived idealizations concerning spatial distribution of resources. Our *N*-habitat models allow us to explore the extent to which local release of *Wolbachia* carriers in a single habitat can elicit propagation to other habitats. As we have seen, propagation depends upon the capacities of each habitat as well as the rates of migration between each pair of habitats.

Another common feature exhibited by PDE-based models of mosquito dispersal is the appearance of diffusion-driven Turing instabilities when the rate of diffusion is varied. While it can be shown analytically that the two-habitat model Eq (7) does not exhibit any Turing instabilities as *m* is varied, such instability can occur in similar “two-cell diffusion” systems. See Section 6.3.2 of [20] for an example.

### Model 2 with multiple habitats

**Fig 9:**
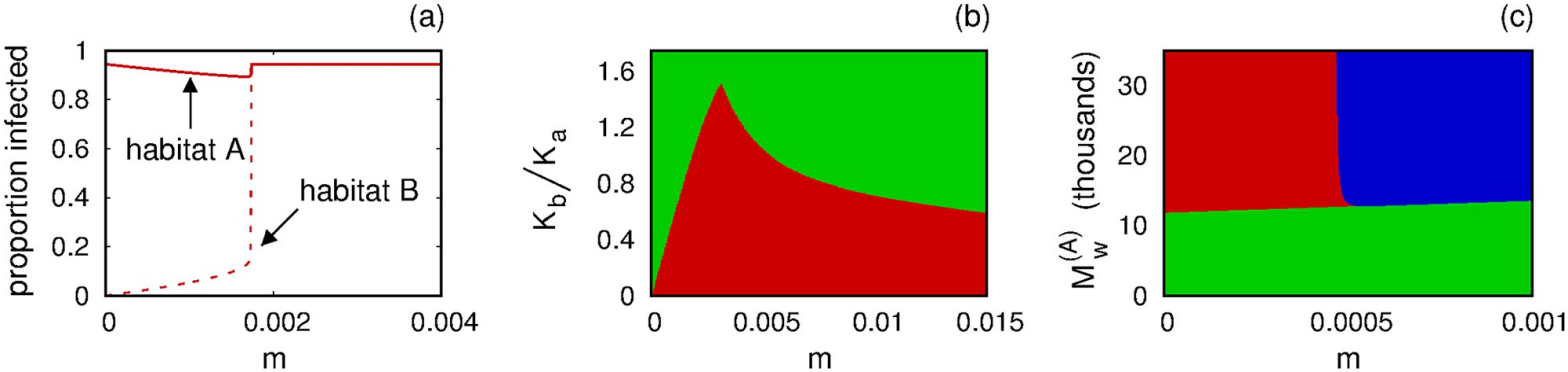
(a) Dependence of steady-state proportion of infected mosquitoes in Habitats A (solid curve) and B (dashed curve) on the migration parameter *m*. (b) Influence of relative habitat size *K*_*b*_/*K*_*a*_ and migration parameter *m* on whether a release of carriers in Habitat A ultimately succeeds (lower, red region) or fails (upper, green region) to establish long-term fixation of carriers in Habitat B. (c) Influence of initial release of carriers in Habitat A and the migration parameter *m* on the eventual outcome: failure to establish carriers in either habitat (lower, green region), fixation of carriers in Habitat A only (upper-left, red region), or fixation of carriers in both habitats (upper-right, blue region). See main text for details, and Tables 3 and 4 for the parameters and initial conditions used to generate these panels.

We felt it important to check whether the predictions made with the multi-habitat adaptation of the (simple) Model 1, are reinforced by analogous simulations with the more biologically-detailed Model 2; see Eq (10). Fig 9 demonstrates that the two-habitat version of Model 2 does, in fact, predict the same behaviors and outcomes as Model 1. The parameter values and initial conditions used to generate the figure are summarized in Tables 3 and 4.

**Table 3:** Baseline values for each fixed parameter used in simulations of the two-habitat adaptation of Model 2. With the exception of *ϕ*_*w*_, these are the same baseline values originally used in [13]. Parameters that were varied, such as *m* and *K*_*b*_, are described in the main text.

Parameter:	*b*_*f*_	*b*_*m*_	*σ*	*ϕ*_*u*_	*ϕ*_*w*_	*v*_*u*_	*v*_*w*_
Value:	0.5	0.5	1.0	13	11 or 13	0.05	0.95
Parameter:	*ψ*	*μ*_*a*_	*μ*_*fu*_	*μ*_*fw*_	*μ*_*mu*_	*μ*_*mw*_	*K*_*a*_
Value:	0.114	0.02	0.0571	0.0633	0.0952	0.0952	2×10^5^

**Table 4:** Baseline values for initial conditions (ICs) used in simulations of the two-habitat adaptation of Model 2; see also Fig 9. Upper rows indicate initial conditions used to generate Fig 9(a) and 9(b). The bottom two rows indicate initial conditions used to generate Fig 9(c).

Variable:	*A*_*u*_	*A*_*w*_	*F*_*u*_	*F*_*w*_	*F*_*pu*_	*F*_*pw*_	*M*_*u*_	*M*_*w*_	*F*_*ps*_
Fig 9(a) and 9(b) Habitat A:	200	300	300	500	100	100	400	0	0
Fig 9(a) and 9(b) Habitat B:	200	0	800	0	100	0	400	0	0
Fig 9(c) Habitat A:	200	0	10	vary	10	0	20	vary	0
Fig 9(c) Habitat B:	200	0	10	0	10	0	20	0	0

Fig 9(a) illustrates how the steady-state proportions of *Wolbachia* carriers in Habitats A and B are influenced by the migration parameter *m*, with all other parameters and initial conditions held fixed, and assuming that Habitats A and B have the same capacities: *K*_*b*_ = *K*_*a*_. For small *m*, carriers are dominant in Habitat A only, as indicated by the solid curve in the upper-left of the figure. As *m* increases from zero, the proportion of carriers in Habitat B (dashed curve) increases gradually at first, before an abrupt transition occurs when *m* reaches a critical threshold (slightly less than *m* = 0.002 for these parameter choices). For larger *m*, the solid and dashed curves coincide, indicating high prevalence of carriers in both habitats. This behavior makes sense: If carriers overtake Habitat A and the rate of migration between Habitats A and B is sufficiently large, then the carriers may also overtake Habitat B.

Fig 9(b) highlights the interplay of relative habitat sizes and migration between habitats. Here, we fix all initial conditions, and all parameters except for the Habitat B capacity *K*_*b*_ and the migration parameter *m*. For each pair (*m*, *K*_*b*_/*K*_*a*_), we compute the proportion of *Wolbachia* carriers in Habitat B at time *t* = 2500, enough time to have achieved approximate steady-state. If this proportion exceeds 0.5, we regard this as “successful” fixation of carriers in Habitat B (lower, red region in the figure). The upper (green) region in the figure indicates a steady-state proportion of carriers less than 0.5. Fig 9(b) also elicits some reasonable predictions: if the migration parameter *m* is small and Habitat B is suitably large relative to Habitat A, then it is impossible to establish carrier fixation in Habitat B even if fixation succeeds in Habitat A.

Fig 9(c) directly addresses our core question: Can a release of carriers in Habitat A lead to fixation of carriers in (i) no habitat, (ii) in Habitat A only, or (iii) in multiple habitats? To generate Fig 9(c), we fixed all initial conditions except for $M_{w}^{\left(A\right)}\left(0\right)$ and $F_{w}^{\left(A\right)}\left(0\right)$, the initial numbers of *Wolbachia*-carrying males and females in Habitat A, respectively. In our simulations, we set $M_{w}^{\left(A\right)}\left(0\right)=F_{w}^{\left(A\right)}\left(0\right)$ in order to simulate a half-female, half-male release of carriers. All parameters except for the migration parameter *m* were fixed. If the initial release of carriers is too small (lower, green region in the figure), then one fails to establish a carrier presence in either habitat. With low migration (small *m*) but a sufficiently large initial release of carriers in Habitat A, one may establish fixation of carriers in Habitat A only (upper-left, red region in the figure). Finally, with suitably large migration (large *m*) and a sufficiently large initial release of carriers in Habitat A, the model predicts fixation of carriers in both habitats (upper-right, blue region in the figure).

Finally, we remark that using the two-habitat version of Model 2, we were also able to replicate the behaviors illustrated in Figs 7 and 8.

## Discussion

In this article, we introduced a framework for modeling mosquito dispersal across distinct, diffusively-coupled habitats, while tracking the prevalence of *Wolbachia*-infected and wild-type subpopulations. Any single-habitat model for mosquito subpopulations can be adapted to a spatially-discrete, multi-habitat model via inclusion of simple diffusive-coupling; we illustrated this using two previously-published models, obtaining qualitatively similar predictions in each case. The rate of diffusion between a given pair of habitats can be estimated in terms of the distance between the habitats and existing data on rates of mosquito movement. We estimated diffusion coefficients using two different types of insect dispersal kernels, together with previously published data on mosquito movement. Because estimates for the mean distance traveled by *Aedes aegypti* vary between studies, years, and locations [24, 25, 30–32], it is important to take this into account when using our spatially-discrete, multi-habitat framework. The choice of dispersal kernel and subsequent estimation of diffusion coefficients should be informed by data from one’s specific geographic region of interest. Such data need not be so fine-grained as to require sophisticated ecological mapping studies; rather, a crude survey of habitat sizes and distances between habitats will suffice.

Our work was motivated by questions of practical importance surrounding the goal of controlling mosquito populations and the spread of vector-borne illnesses. Can the deliberate introduction of *Wolbachia* carriers in one habitat achieve a persistent presence of these carriers? Models 1 and 2 suggest that the answer is ‘yes’, provided that the number of released carriers is sufficiently large. Of course, this sensible prediction is not novel—it appears in the articles surveying the dynamics of the single-habitat versions of these models [2, 13]. In the present study, our primary questions necessitated generalization of previous models to multiple habitats: Can a release of *Wolbachia* carriers in one habitat elicit a sustained presence of these carriers in other nearby habitats and, if so, what factors determine whether this occurs? Our simulations indicated that a release of carriers in one habitat (say Habitat A) can yield several possible outcomes (see also Fig 9c):

- Outcome 1: failure to establish a carrier presence in any habitat (e.g., if the size of the initial release is too small);
- Outcome 2: sustained presence of carriers in Habitat A but no other habitats, (e.g., if other habitats are too distant from Habitat A); and
- Outcome 3: sustained presence of carriers in Habitat A and some other nearby habitats.

According to the multi-habitat version of Model 1, one way to achieve Outcome 3 is to release enough carriers in every habitat to achieve fixation in each individual habitat without even considering diffusive coupling between habitats. Of course, this might require significant effort with respect to the breeding and release of infected mosquitoes. However, our numerical simulations indicate that given the right circumstances, fixation of carriers in multiple habitats can be achieved by releasing carriers in a single habitat and relying upon diffusive coupling to spread *Wolbachia* into other territories.

If one seeks to achieve Outcome 3 (see preceding paragraph), it is natural to ask how and where to release *Wolbachia* carriers. The answer depends upon several factors, including the carrying capacities of individual habitats and the diffusion coefficients. If Habitat A is half the size of Habitat B, then one expects that sustained infection in Habitat A can be achieved by a smaller initial release of carriers than would be needed to accomplish the same in Habitat B. While this may seem to suggest that releasing infected mosquitoes in small, easily conquered habitats is a good course of action, the rate of migration of *Wolbachia* carriers from Habitat A to Habitat B may be insufficient to establish fixation of carriers in Habitat B. Success depends upon whether the proportion of carriers in Habitat B ever exceeds some threshold. It may be more effective to perform a larger initial release of carriers in Habitat B, enough to achieve fixation in this larger habitat. Subsequently, the rate of migration of carriers from B to A may be sufficient to achieve infection fixation in Habitat A as well.

Another important consideration regarding the initial release of infected mosquitoes is the sex of the mosquitoes. *Wolbachia*-based biological control relies upon the cytoplasmic incompatibility resulting from crossing male and female mosquitoes of opposite carrier status, and can be exploited for the control of various insect species [33]. Assuming that cytoplasmic incompatibility is unidirectional in the sense that it requires mating infected males with uninfected females, the initial release should consist only of infected males. (Another advantage of releasing only males is the fact that they do not bite.) However, for genetic approaches towards mosquito control, it is possible that higher success can be achieved through release of females [34].

Following up on the preceding paragraph, one important area of future study is the importance of sex ratios on long-term outcomes. The relative abundance of vegetation and standing water in a habitat may influence the effective carrying capacities for female and male mosquitoes. This may well influence strategies for release of carriers. Of the two population models we tested in this article, neither Model 1 nor Model 2 has the flexibility to directly specify separate carrying capacities for females and males—Model 1 does not distinguish between sexes, and Model 2 incorporates a capacity only for the aquatic stage. We did, however, perform some additional simulations with Model 2 in which the carrying capacities for females and males were adjusted indirectly through modification of the death rate constants:*μ*_*fu*_ and *μ*_*fw*_ for females and *μ*_*mu*_ and *μ*_*mw*_ for males. We simulated a release of carrier females in Habitat A. If the death rate of females in Habitat A is low (which indirectly sets a high carrying capacity for females), then the infection spreads to Habitat B as expected. However, if the death rate of females is high in Habitat A (effectively setting a low carrying capacity for females), then one fails to capture either habitat. Adapting Model 2 to afford more direct control over adult-stage carrying capacities for each sex is beyond the scope of the present study, but merits future attention.

In closing, we remark that our use of dispersal kernels and spatially-discrete diffusion models can be applied in a variety of other contexts. *Wolbachia* has been proposed as a means for insect pest control for species other than mosquitoes (see for example [33]). Other methods of pest control (e.g., gene drives) can also be explored using methods described in the present article. Moreover, the use of dispersal and redistribution kernels is by no means specific to insects; see, for example, the text of [35]. While texts such that one formulate animal movement models as advection-reaction-diffusion PDEs, here we have preferred to model landscapes as a network of discrete, distinct habitats. By adopting this “coarser” view of the landscape, we avoid the need for fine description of spatially heterogeneous resource density.

## Supporting information

S1 Appendix Estimating *m* from a probability density function. Derives Eq (17).
(ZIP)

S1 Fig (TIF)

S2 Fig (TIF)
