# Supplementary material for: Efficacy of Wolbachia-based mosquito control: Predictions of a spatially discrete mathematical model
Source: PLoS One. 2024 Mar 4;19(3):e0297964. doi: 10.1371/journal.pone.0297964 (PMC10911593; doi:10.1371/journal.pone.0297964)
Supplement: S1 Appendix — Derives Eq (17). (ZIP) [file pone.0297964.s001.zip › SI.pdf]

Supporting Information for "Efficacy of *Wolbachia*-based mosquito control: Predictions of a spatially discrete mathematical model"

David Dye[1], John W. Cain[1*]

1 Department of Mathematics, Harvard University, Cambridge, MA

* jcain2@math.harvard.edu

Appendix: Estimating m From a Probability Density Function

Here, we derive Eq. (17) from the main text, obtaining an estimate for m for any radially-symmetric probability density function (PDF). For convenient reference, we restate the equation

$$m(x^*) = \frac{1}{2\pi t} \int_0^{2\pi} \int_0^{r^*} \frac{p(r\cos\theta + x^*, r\sin\theta)}{\sqrt{(r\cos\theta + x^*)^2 + (r\sin\theta)^2}}\, r\; dr\; d\theta \tag{1}$$

and recall that x^* represents the distance separating the centers of two circular habitats of radius r^*. The variable t is the time elapsed during the experimental period used to estimate the PDF, denoted by p.

The migration parameter m represents the proportion of the mosquito population in a habitat that moves from that habitat to another per unit time. A dispersal kernel is the PDF describing the probability that a mosquito can be found some distance away from the distribution's center. Dispersal kernels can be approximated from MRR data, by measuring mosquito displacement from a release point after some amount of time t has elapsed. The PDF can be used to compute the probability P that a mosquito from one habitat is located in the other habitat after a time t has elapsed. Naturally, the probability that a mosquito has traveled a distance x depends upon t. Over short time scales, perhaps the simplest assumption regarding the temporal evolution of a dispersal kernel is that of linear radial growth; see [1], particularly Figure 1(a) of that article. With this assumption, the relationship between $m(x^*)$, t, and the probability $P(x^*)$ is given by

$$m(x^*) = \frac{P(x^*)}{t}. \tag{2}$$

To use Eq. (2), $p(x, y)$ must be converted to $P(x^*)$, the probability of a mosquito from one habitat being located in the region of the second habitat. Assume that mosquitoes are dispersed radially from the center of each habitat as described by the PDF. Then, there will be some radius r^* encompassing a proportion q of that habitat's mosquito population.

The dispersal kernel PDF is the probability density function of being some distance away from the distribution's center in any direction. However, a habitat is located at some specific location away. Therefore, the dispersal kernel PDF must be converted to a dispersal location kernel PDF (denoted by p_L), which is done by dividing by $2\pi r$ [2]. In Cartesian coordinates,

$$p_L(x, y) = \frac{p(x, y)}{2\pi\sqrt{x^2 + y^2}}. \tag{3}$$

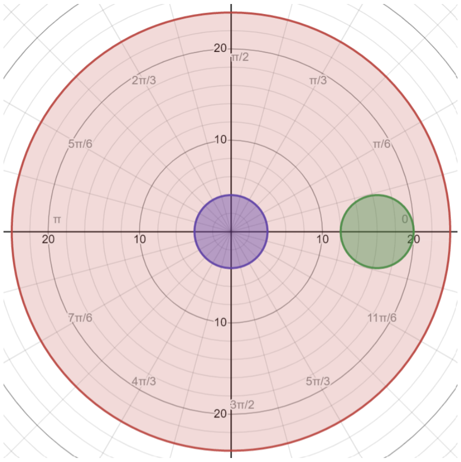

Fig 1. The probability of a mosquito being in the green region of Habitat B is given by the dispersal location kernel integrated over the green region. The purple region represents the mosquito's home habitat, Habitat A. The distance separating the centers of the habitats is x^*, and the radii of the habitats are both r^*.

Now, it is possible to integrate over a specific region in 2D space. Define an x, y plane with the center of Habitat A at the origin. Then, the center of Habitat A's mosquito distribution is also at the origin. Let Habitat B be a circular region a distance of x^* away from Habitat A along the x axis. Because p_L is radially symmetric, the axes of the coordinate plane and the location of Habitat B with respect to them are arbitrary, as long as the distance from Habitat A to Habitat B is always x^*. See Fig. 1.

This is enough information to integrate $p_L(x, y)$ from Habitat A over the region of Habitat B, which results in the probability of a Habitat A mosquito being in Habitat B. The integral is given by the following:

$$P(x^*) = \frac{1}{2\pi} \int_{x^*-r^*}^{x^*+r^*} \int_{-\sqrt{(r^*)^2-(x-x^*)^2}}^{\sqrt{(r^*)^2-(x-x^*)^2}} \frac{p(x,y)}{\sqrt{x^2+y^2}} \, dy \, dx. \tag{4}$$

Shifting coordinates by substituting $u = x - x^*$,

$$P(x^*) = \frac{1}{2\pi} \int_{-r^*}^{r^*} \int_{-\sqrt{(r^*)^2-(u)^2}}^{\sqrt{(r^*)^2-(u)^2}} \frac{p(u+x^*,y)}{\sqrt{(u+x^*)^2+y^2}} \, dy \, du. \tag{5}$$

Graphically, the last step shifted the coordinate plane such that Habitat B is centered around the origin. This is shown in Fig. 2.

The bounds of integration of Eq. (5) are nicer if the integral is converted to polar coordinates. With $y = r\sin\theta$ and $u = r\cos\theta$, one obtains

$$P(x^*) = \frac{1}{2\pi} \int_0^{2\pi} \int_0^{r^*} \frac{p(r\cos\theta + x^*, r\sin\theta)}{\sqrt{(r\cos\theta + x^*)^2 + (r\sin\theta)^2}} \, r \, dr \, d\theta. \tag{6}$$

Eq. (6) gives the probability that a mosquito will be in an idealized circular habitat of radius r^* a distance x^* away from the center of its original habitat. It is true for any radially-symmetric dispersal kernel $p(x, y)$. Substituting Eq. (6) into Eq. (2) yields

$$m(x^*) = \frac{1}{2\pi t} \int_0^{2\pi} \int_0^{r^*} \frac{p(r\cos\theta + x^*, r\sin\theta)}{\sqrt{(r\cos\theta + x^*)^2 + (r\sin\theta)^2}} \, r \, dr \, d\theta, \tag{7}$$

the formula for m appearing as Eq. (17) in the main text.

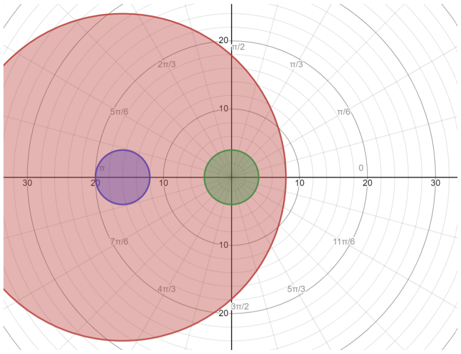

Fig 2. The coordinate plane has been shifted so Habitat B is at the origin.

References

1. Hallatschek O, Fisher DS. Acceleration of evolutionary spread by long-range dispersal. Proceedings of the National Academy of Sciences 2014; 111(46):E4911–E4919.

2. Nathan R, Klein E, Robledo-Arnuncio JJ, Revilla E. Dispersal kernels: review. Oxford: Oxford University Press; 2012.
