# Supplementary figures and images for: Efficacy of Wolbachia-based mosquito control: Predictions of a spatially discrete mathematical model
Source: PLoS One. 2024 Mar 4;19(3):e0297964. doi: 10.1371/journal.pone.0297964 (PMC10911593; doi:10.1371/journal.pone.0297964)

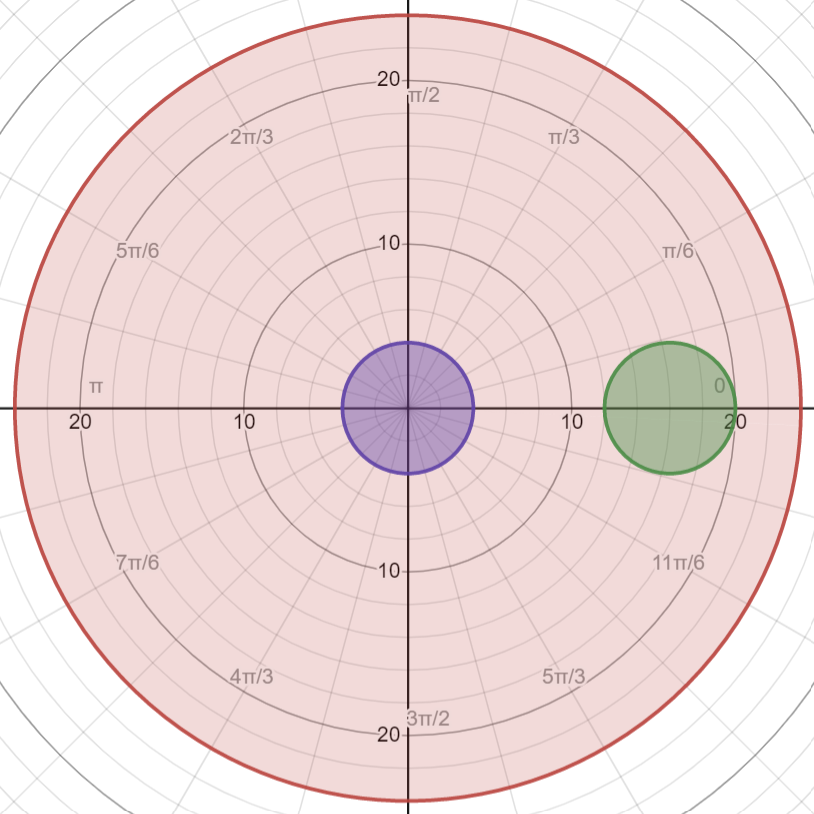

Supplement: S1 Fig — (TIF) [file pone.0297964.s002.tif]

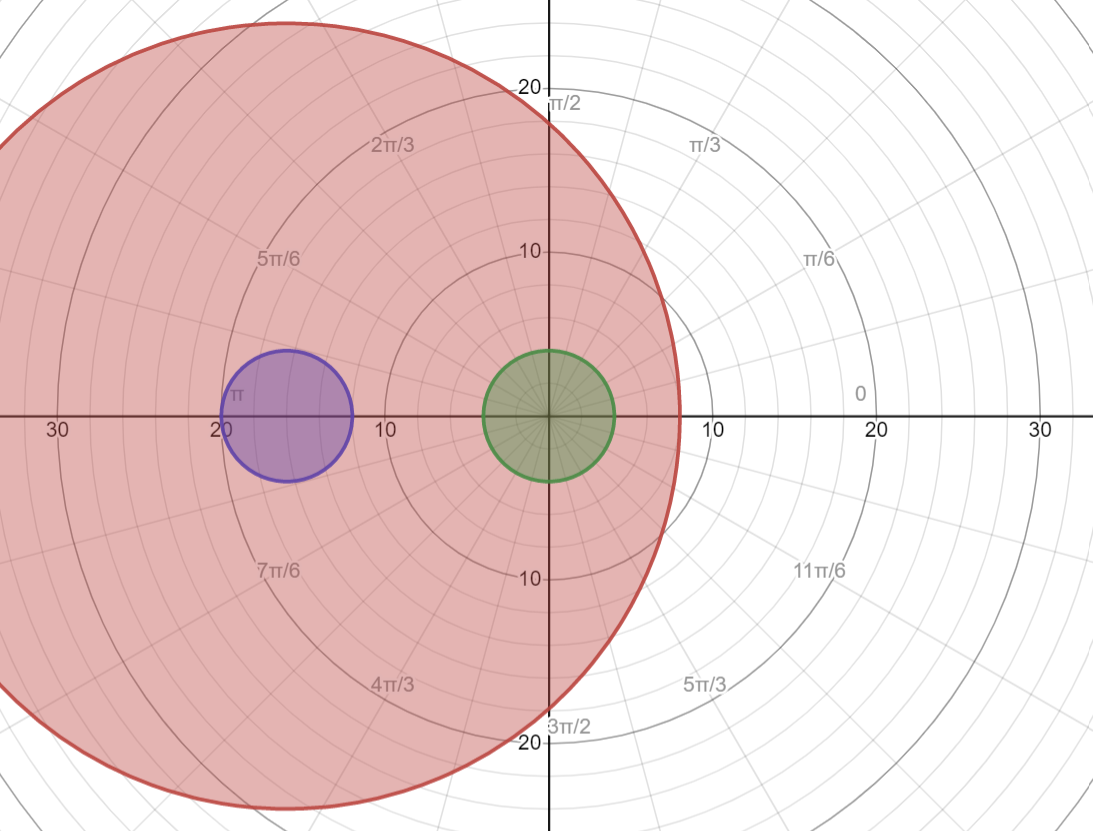

Supplement: S2 Fig — (TIF) [file pone.0297964.s003.tif]
